# Enhanced UHPLC-MS/MS screening of selective androgen receptor modulators following urine hydrolysis

**DOI:** 10.1016/j.mex.2020.100926

**Published:** 2020-05-21

**Authors:** Anna Gadaj, Emiliano Ventura, Jim Healy, Francesco Botrè, Saskia S. Sterk, Tom Buckley, Mark H. Mooney

**Affiliations:** a Institute for Global Food Security, School of Biological Sciences, Queen's University Belfast, BT9 5DL, United Kingdom; b Chemical and Immunodiagnostic Sciences Branch, Veterinary Sciences Division, Agri-Food & Biosciences Institute (AFBI), Stoney Road, Belfast BT4 3SD, United Kingdom; c Laboratory, Irish Greyhound Board, Limerick Greyhound Stadium, Ireland; d Applied Science Department, Limerick Institute of Technology, Moylish, Limerick, Ireland; e Laboratorio Antidoping, Federazione Medico Sportiva Italiana, Italy; f Wageningen Food Safety Research, Wageningen University & Research, European Union Reference Laboratory, Wageningen, the Netherlands; g Irish Diagnostic Laboratory Services Ltd., Johnstown, W91 RH93, Co. Kildare, Ireland

**Keywords:** SARMs, Urine, Hydrolysis, UHPLC-MS/MS, Doping analysis, Food safety

## Abstract

Selective androgen receptor modulators (SARMs) represent non-steroidal agents commonly abused in human and animal (i.e. equine, canine) sports, with potential for further misuse as growth promoting agents in livestock-based farming. As a direct response to the real and possible implications of illicit application in both sport as well as food production systems, this study incorporated enzymatic hydrolysis (β-glucuronidase/arylsulfatase) into a previously established protocol while maintaining the minimal volume (200 µL) of urine sample required to detect SARMs encompassing various pharmacophores in urine from a range of species (i.e. equine, bovine, human, canine and rodent). The newly presented semi-quantitative UHPLC-MS/MS-based assay is shown to be fit-for-purpose, being rapid and offering high-throughput, with validation findings fulfilling criteria stipulated within relevant doping and food control legislation.

• CCβ values determined at 1 ng mL^−1^ for majority of analytes.
• Deconjugation step included in the method led to significantly increased relative abundance of ostarine in analysed incurred urine samples demonstrating the requirement for hydrolysis to detect a total form of emerging SARMs.
• Assay amenable for use within routine testing to ensure fair play in animal and human sports and that animal-derived food is free from contamination with SARM residues.

## Graphical abstract

**Figure:**
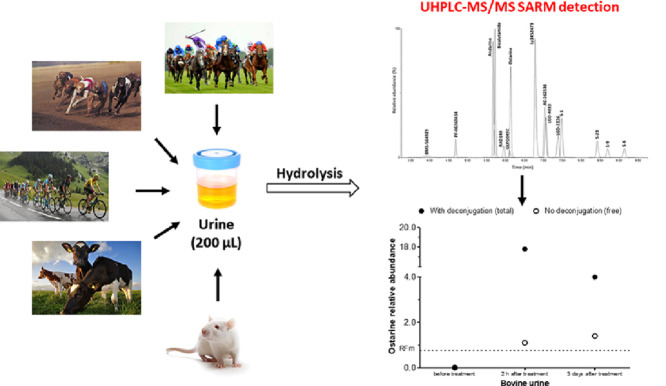

Specifications Table

**Table:** 

Subject Area	Chemistry
More specific subject area	Analytical chemistry
Method name	UHPLC-MS/MS-based screening of SARMs following urine hydrolysis
Name and reference of original method	E. Ventura, A. Gadaj, G. Monteith, A. Ripoche, J. Healy, F. Botrè, S. S. Sterk, T. Buckley and M. H. Mooney, Journal of Chromatography A, 2019, 1600, 183-196.
Resource availability	AC-262536 (P/N 96443-25MG, Sigma-Aldrich, Dublin, Ireland), andarine (S-4, P/N 78986-25MG, Sigma-Aldrich, Dublin, Ireland), bicalutamide (P/N PHR-1678-1G, Sigma-Aldrich, Dublin, Ireland), BMS-564929 (10 mM solution in DMSO, P/N HV-12111, MedChem Express, Sollentuna, Sweden), GLPG0492 (10 mM solution in DMSO, P/N HY-18102, MedChem Express, Sollentuna, Sweden), LGD-2226 (P/N 07682-25MG, Sigma-Aldrich, Dublin, Ireland), LGD-4033 (P/N CAY9002046-50mg, Cambridge Bioscience Ltd., Cambridge, UK), Ly2452473 (P/N CDS025139-50MG, Sigma-Aldrich, Dublin, Ireland), ostarine (S-22, P/N MK-2866, Cambridge Bioscience Ltd., Cambridge, UK), PF-06260414 (P/N PZ0343-5MG, Sigma-Aldrich, Dublin, Ireland), RAD140 (P/N CAY18773-1mg, Cambridge Bioscience Ltd., Cambridge, UK), S-1 (P/N 68114-25MG, Sigma-Aldrich, Dublin, Ireland), S-6 (P/N 79260-25MG, Sigma-Aldrich, Dublin, Ireland), S-9 (P/N D289535, Toronto Research Chemicals, Toronto, Canada), S-23 (P/N 55939-25MG, Sigma-Aldrich, Dublin, Ireland), bicalutamide-D_4_ (P/N B382002, Toronto Research Chemicals, Toronto, Canada), S-1-D_4_ (P/N D289532, Toronto Research Chemicals, Toronto, Canada); ultra-pure water (18.2 MOhm, generated in house using a Millipore (Cork, Ireland) water purification system), ethanol (EtOH) and dimethyl sulfoxide (DMSO) (both ACS reagent grade, Sigma-Aldrich, Dublin, Ireland), methanol (MeOH) and acetonitrile (MeCN) (both Chromasolv™ LC-MS grade, Honeywell, VWR International, Dublin, Ireland), acetonitrile-D (MeCN-D, 99.5%, Sigma-Aldrich, Dublin, Ireland), ammonium hydroxide solution, ≥25% (NH_4_OH) and acetic acid (CH_3_COOH) (both eluent additives for LC-MS, Honeywell, VWR International, Dublin, Ireland), *tert*-butyl methyl ether (TBME, LiChrosolv® LC grade, Sigma-Aldrich, Dublin, Ireland), sodium acetate (powder, BioReagent grade, Sigma-Aldrich, Dublin, Ireland), β-glucuronidase/arylsulfatase from *Helix pomatia* (stabilised saline solution, Roche, P/N 10127698001, Sigma-Aldrich, Dublin, Ireland); PAL-USG (CAT) pocket refractometer (Atago, Tokyo, Japan), SafeSeal polypropylene micro tubes (2 mL, Sarstedt, Nümbrecht, Germany), Hettich Micro 200R centrifuge (Davidson & Hardy, Belfast, UK), DVX-2500 multi-tube vortexer (VWR International, Dublin, Ireland), Grant GLS400 water bath with shaking (Davidson & Hardy, Belfast, UK), centrifuge filters 0.22 µm PTFE 750 µL centrifuge filters 0.22 µm PTFE (P/N F2517-9, Thermo Fisher Scientific, Hemel Hempstead, UK), Turbovap® LV evaporator (Caliper Life Sciences, Mountain View, USA); Waters Acquity I-Class UPLC® system (Milford, MA, USA) coupled to a Waters Xevo® TQ-MS triple quadrupole mass analyser (Manchester, UK) controlled by MassLynx™ software (TargetLynx™ software for data processing, Waters), Luna® Omega Polar C18 (100 × 2.1 mm, 100 Å, 1.6 µm, P/N 00D-4748-AN, Phenomenex, Cheshire, UK), KrudKatcher™ Ultra HPLC in-line filter (P/N AF0-8497, Phenomenex, Cheshire, UK) .

## Method details

### Background

Selective androgen receptor modulators (SARMs) encompass a class of drugs with diverse non-steroidal pharmacophores reported to be widely abused in human and animal sports through their oral bioavailability and biological potency which is facilitated by their widespread availability [1]. SARM compounds have potential to find use in livestock-based food production [2] systems seeking growth promoting and feed efficiency benefits. Hormonal acting substances are banned within farming in the EU since 1988 [3], and assays with capability to detect potential SARMs’ abuse are therefore needed to aid the effective enforcement of prohibition [4]. In this regard a range of methods in respect to the LC-MS/MS analysis of SARM residues in urine have been reported (recently reviewed by Ventura et al. [5]). Additionally investigations into the metabolic fate of selected SARM compounds in various species (e.g. equine, bovine, human) have revealed that intact molecules and/or their respective generated phase I SARM metabolites undergo phase II conjugation (i.e. with glucuronic acid and/or sulphate moieties) [1,[6], [7], [8]. However, variability in the range of different SARM pharmacophores and also in the pattern of interspecies metabolic biotransformation, is compounded by the lack of firm data in the scientific literature arising from drug elimination studies as well as an absence of reference materials and standards for associated biotransformation products. Consequently, implementation into routine urine analysis of procedures employing an enzymatic deconjugation step (cleavage of both glucuronide and sulphate conjugates) using e.g. *Helix pomatia* digestive juice [7,^9^,10] is recommended providing for superior detection windows *via* the indirect detection of the corresponding aglycones of SARMs and/or their metabolites. Our group reported previously [5] a semi-quantitative method to monitor the misuse of 15 SARM compounds belonging to nine different families, in urine matrices from a range of species (equine, canine, human, bovine and rodent). Briefly, SARM residues were extracted from urine (200 µL) with TBME without further clean-up and analysed by UHPLC-MS/MS. A 12 min gradient separation was carried out on a Luna Omega Polar C18 column, employing water and methanol, both containing 0.1% acetic acid (*v/v*), as mobile phases. Validation was performed according to the EU Commission Decision 2002/657/EC criteria and European Union Reference Laboratories for Residues (EU-RLs) guidelines with CCβ values determined at 1 ng mL^−1^, excluding andarine (2 ng mL^−1^) and BMS-564929 (5 ng mL^−1^), in all species. The current study therefore seeks to incorporate enzymatic hydrolysis into a previously reported screening protocol [5] to deliver a reliable and effective tool to reveal illicit SARM use in urine from animal and human sport animals as well as food-based livestock that can be adopted and implemented in various residue monitoring programmes.

### Reagents

All reagents used in this research were of analytical grade or better (*Resource availability* section). β-glucuronidase/arylsulfatase from *Helix pomatia* activity as per manufacturer's information: the β-glucuronidase (*EC 3.2.1.31*) - 4.5 U mL^−1^, equivalent to 5.5 phenolphthalein U mL^−1^ or 100,000 Fishman units, pH 4.5, 25°C, the arylsulfatase (*EC 3.1.6.1*) - 14 U mL^−1^, equivalent to 2.6 phenolphthalein U mL^−1^ or 800,000 Roy units, pH 6.2, 25°C. Sources and preparation of all standards and solutions used in the current assay are as detailed elsewhere [5]. Briefly, all individual standard stock solutions were prepared at a concentration of 1 mg mL^−1^ in an appropriate solvent: DMSO (AC-252636, andarine (S-4), LGD-2226, LGD-4033, Ly2452473, PF-06260414, RAD140 and S-23), MeCN (bicalutamide, ostarine (S-22) and S-1), EtOH (S-6 and S-9). 10 mmol L^−1^ standard solutions in DMSO of BMS-564929 and GLPG0492 were diluted with DMSO to give a concentration of 1 mg mL^−1^, respectively. Internal standards stock solutions were prepared at a concentration of 1 mg mL^−1^ in MeCN-D. Intermediate mixed standard solutions were prepared at the following concentrations: 20 / 40 (andarine) / 100 (BMS-564929), 1 / 2 (andarine) / 5 (BMS-564929) and 0.1 / 0.2 (andarine) / 0.5 (BMS-564929) µg mL^−1^ in MeCN by serial dilutions. Working quality control standard solution at a concentration of 10 / 20 (andarine) / 50 (BMS-564929) ng mL^−1^ was prepared in MeCN. Intermediate internal standard mix solutions were prepared at 20 and 1 µg mL^−1^, respectively, using MeCN-D as the diluent. A working internal standard mix solution was prepared at 50 ng mL^−1^ in MeCN-D. All standards and internal standards stock solutions were stored at -20°C

### Analysis of SARM residues in urine by UHPLC-MS/MS

Urine samples were stored at -80°C and centrifuged at 4,500 × *g* for 10 min at 4°C prior to analysis. Urinary specific gravity was assessed and pH adjusted as required with acetic acid to 5.5 ± 0.1 and 200 µL aliquots fortified with 20 µL of a 50 ng mL^−1^ internal standard mix. After 15 min, 0.1 mol L^−1^ acetate buffer pH 5.5 (200 µL) was added to each sample and vortexed for 10 s, with 50 µL of β-glucuronidase/arylsulfatase diluted with H_2_O (1:5, *v/v*) subsequently added. Samples were vortexed again for 10 s, incubated in a water bath (with shaking) at 55°C for 1 h and then allowed to cool (*ca.* 10 min). Following addition of 50 mmol L^−1^ aqueous NH_4_OH pH 10.5 (200 µL) to each sample, tube contents were vortexed for 60 s and 1.5 mL of TBME added. Samples were vortexed for 15 min, centrifuged at 24,400 × *g* for 10 minutes at 4°C, and supernatants transferred into 2 mL micro tubes and evaporated to dryness under nitrogen (≤5 Bar) at 40°C (Turbovap® LV system). Dried samples were reconstituted in 100 µL H_2_O:MeCN (4:1, *v/v*) by vortexing (5 min) and extracts filtered at 10,840 × *g* for 2 min at 15°C prior to injection (9 µL) onto a UHPLC-MS/MS system.

**Fig. 1:**
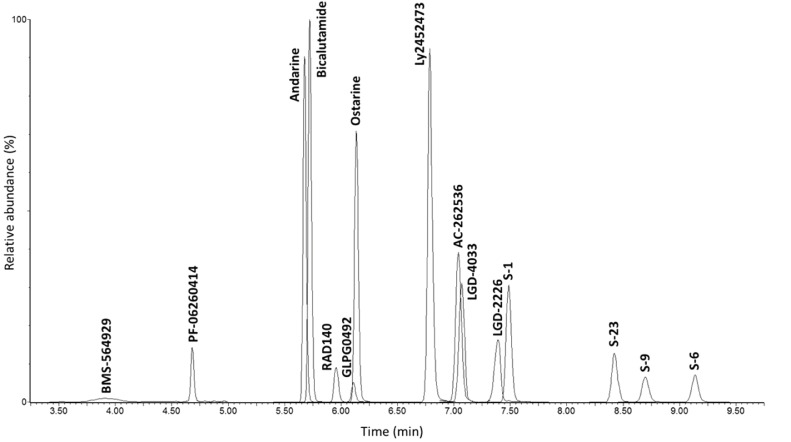
Overlay of representative extracted ion chromatograms of a blank equine urine fortified at screening target concentration (C_val_) with SARMs of interest.

Procedures for analysis of selected SARM compounds by UHPLC-MS/MS were previously optimised by our group [5]. Nevertheless, the current chromatographic separation was employed following some modifications [11,12], with conditions specific to the presented method summarised in Tables 1 and 2. A typical chromatogram is shown in Fig. 1 with all target SARM compounds separated during the first 9.45 min of chromatographic analysis.

**Table 1:** Analytical platform and respective conditions.

**Waters Acquity I-Class UPLC®**	
Column	Luna® Omega Polar C18 (100 × 2.1 mm, 100 Å, 1.6 µm) supplied with KrudKatcher™ Ultra HPLC in-line filter, 45°C
Mobile phase A	0.1% (*v/v*) CH_3_COOH in H_2_O
Mobile phase B	0.1% (*v/v*) CH_3_COOH in MeOH
Flow rate	0.40 mL min^−1^
Run time	14 min
Injection volume	9 µL
Gradient profile	(1) 0.00 min 20% B, (2) 0.50 min 20% B, (3) 4.75 min 60% B, (4) 10.50 min 67.5% B, (5) 11.00 min 99% B, (6) 12.00 min 99% B, (7) 12.10 min 20.0% B, (8) 14.00 min 20% B
Flow diverted to waste	11.00 - 13.50 min
Needle wash	H_2_O:MeOH (1:1, *v/v*)
Needle purge	H_2_O:MeOH (4:1, *v/v*)
Seal wash	% % %%%%%%%% H_2_O:MeOH (95:5, *v/v*)
**Waters Xevo® TQ-MS**	
Capillary voltage	2.50 kV (ESI+), 1.00 kV (ESI-)
Source temperature	120°C
Desolvation gas temperature	550°C
Desolvation gas flow	900 L h^−1^
Collision gas flow	0.15 mL min^−1^

**Table 2:** UHPLC-MS/MS conditions for urine samples.

No	Analyte	Formula	T_R_^a^ (min)	Transition (*m/z*)	Dwell time (s)	Cone (V)	CE^b^ (eV)	SRM window^c^	ESI polarity	IS^d^
1	AC-262536	C_18_H_18_N_2_O	7.04	279.2 > 195.0^e^	0.025	36	22	1	+	N/A
				279.2 > 169.1			24			
				279.2 > 93.0			22			
2	Andarine (S-4)	C_19_H_18_F_3_N_3_O_6_	5.68	440.2 > 150.0^e^	0.010	30	30	15	-	Bicalutamide-D_4_
				440.2 > 261.1			20			
				440.2 > 205.0			34			
				440.2 > 107.0			46			
3	Bicalutamide	C_18_H_14_F_4_N_2_O_4_S	5.72	429.2 > 255.0^e^	0.007	24	16	13	-	Bicalutamide-D_4_
				429.2 > 185.0			46			
				429.2 > 173.0			24			
4	BMS-564929	C_14_H_12_ClN_3_O_3_	3.93	306.1 > 96.0^e^	0.350	30	16	3	+	N/A
				306.1 > 86.1			24			
				306.1 > 278.1			14			
5	GLPG0492	C_19_H_14_F_3_N_3_O_3_	6.11	390.2 > 118.0^e^	0.017	34	44	5	+	N/A
				390.2 > 360.2			20			
				390.2 > 91.0			38			
6	LGD-2226	C_14_H_9_F_9_N_2O_	7.39	393.1 > 241.1^e^	0.015	60	38	6	+	N/A
				393.1 > 223.0			52			
				393.1 > 375.1			32			
				393.9 > 203.1			56			
7	LGD-4033	C_14_H_12_F_6_N_2_O	7.07	337.1 > 267.2^e^	0.025	28	10	8	-	N/A
				337.1 > 170.0			24			
				337.1 > 239.1			24			
8	Ly2452473	C_22_H_22_N_4_O_2_	6.79	375.2 > 272.1^e^	0.040	30	20	4	+	N/A
				375.2 > 289.2			18			
				375.2 > 92.8			38			
				375.2 > 180.0			38			
9	Ostarine (S-22)	C_19_H_14_F_3_N_3_O_3_	6.14	388.1 > 118.0^e^	0.017	30	20	9	-	Bicalutamide-D_4_
				388.1 > 269.1			18			
				388.1 > 90.0			54			
10	PF-06260414	C_14_H_14_N_4_O_2_S	4.68	303.1 > 232.1^e^	0.040	36	24	2	+	N/A
				303.1 > 168.2			36			
				303.1 > 210.1			26			
11	RAD140	C_20_H_16_ClN_5_O_2_	5.96	394.1 > 223.1^e^	0.005	20	10	7	+	N/A
				394.1 > 170.1			30			
				394.1 > 205.1			20			
12	S-1	C_17_H_14_F_4_N_2_O_5_	7.49	401.1 > 261.1^e^	0.025	35	20	10	-	S-1-D_4_
				401.1 > 205.0			26			
				401.1 > 111.0			24			
				401.1 > 289.1			20			
13	S-6	C_17_H_13_ClF_4_N_2_O_5_	9.14	435.1 > 145.0^e^	0.060	30	25	14	-	S-1-D_4_
				435.1 > 289.1			20			
				435.1 > 261.1			20			
				435.1 > 205.0			30			
14	S-9	C_17_H_14_ClF_3_N_2_O_5_	8.69	417.2 > 261.2^e^	0.060	30	20	12	-	S-1-D_4_
				417.2 > 127.0			28			
				417.2 > 205.0			30			
15	S-23	C_18_H_13_ClF_4_N_2_O_3_	8.42	415.2 > 145.0^e^	0.060	30	24	11	-	S-1-D_4_
				415.2 > 185.0			34			
				415.2 > 269.1			18			
16	Bicalutamide-D_4_	C_18_H_10_D_4_F_4_N_2_O_4_S	5.71	433.2 > 255.1	0.007	26	14	13	-	N/A
17	S-1-D_4_	C_17_H_10_D_4_F_4_N_2_O_5_	7.45	405.2 > 261.1	0.025	34	20	10	-	N/A

a T_R_, retention time.

b CE, collision energy.

c SRM 1 (6.80-7.40 min); SRM 2 (4.40-5.00 min); SRM 3 (3.40-4.50 min); SRM 4 (6.50-7.10 min); SRM 5 (5.95-6.35 min); SRM 6 (7.15-7.75 min); SRM 7 (5.70-6.30 min); SRM 8 (6.80-7.40 min); SRM 9 (5.90-6.50 min); SRM 10 (7.25-7.85 min); SRM 11 (8.20-8.80 min); SRM 12 (8.45-9.05 min); SRM 13 (5.45-6.05 min); SRM 14 (8.85-9.45 min); SRM 15 (5.40-6.00 min).

d Internal standard. The response factor was obtained as a ratio between analyte peak area and internal standard peak area, in the case of the other SARMs, peak area was used as the response.

e Diagnostic ion.

### Extracted urine screen positive and recovery control checks

Pooled negative urine (*n* = 10) was used for quality control (QC) purposes as described previously [5]. Briefly, extracted matrix screen positive controls were prepared by fortifying negative QC samples (*n* = 3) prior to extraction with 20 µL of quality control standard solution (10 / 20 (andarine) / 50 (BMS-564929) ng mL^−1^) to give a screening target concentration in urine for all analytes of 1 ng mL^−1^, with the exception of andarine (2 ng mL^−1^) and BMS-564929 (5 ng mL^−1^). Additional negative samples (*n* = 2) were spiked post-extraction with QC standard solution (20 µL) to monitor for analyte loss during extraction. Results from on-going QC samples (i.e. negative, screen positive and recovery controls) are being recorded to verify performance reliability and robustness of the assay.

## Additional information

### Method optimization

This study aimed to incorporate an enzymatic urine hydrolysis step into a previously established UHPLC-MS/MS protocol [5,^11^,12] whilst maintaining the minimal volume (200 µL) of sample required. Enzymatic methods are commonly used as a hydrolysis approach being generally more specific with procedures performed in milder conditions in comparison to chemical (acid or alkaline) hydrolysis, ensuring the stability of target analytes and/or sample integrity. However, a number of factors impact efficiency of residue deconjugation namely temperature, time of incubation, pH and amount of enzyme [13]. Due to the lack of respective SARM conjugate standards, β-glucuronidase/arylsulfatase from *Helix pomatia* was used as per manufacturer's instructions. The sole parameter assessed during method development was the applicability of 0.1 mol L^−1^ carbonate buffer (pH 9.5) or 50 mmol L^−1^ aqueous NH_4_OH (pH 10.5) to elevate pH from pH 5.5 used during the enzymatic hydrolysis process, with the later chosen providing satisfactory recovery for all SARM compounds of interest (Supplementary data - Fig. S1).

### Method validation

The current assay was validated with regard to selectivity, specificity, detection capability (CCβ), sensitivity, limit of detection (LOD), absolute recovery and matrix effects, according to respective EU legislation [14,15] to demonstrate compliance with required performance criteria. Validation was carried out at the screening target concentration (C_val_) of 1 ng mL^−1^ excluding andarine (2 ng mL^−1^) and BMS-564929 (5 ng mL^−1^) as detailed in Ventura et al. [5].

#### Selectivity, specificity, and matrix effect studies

**Fig. 2:**
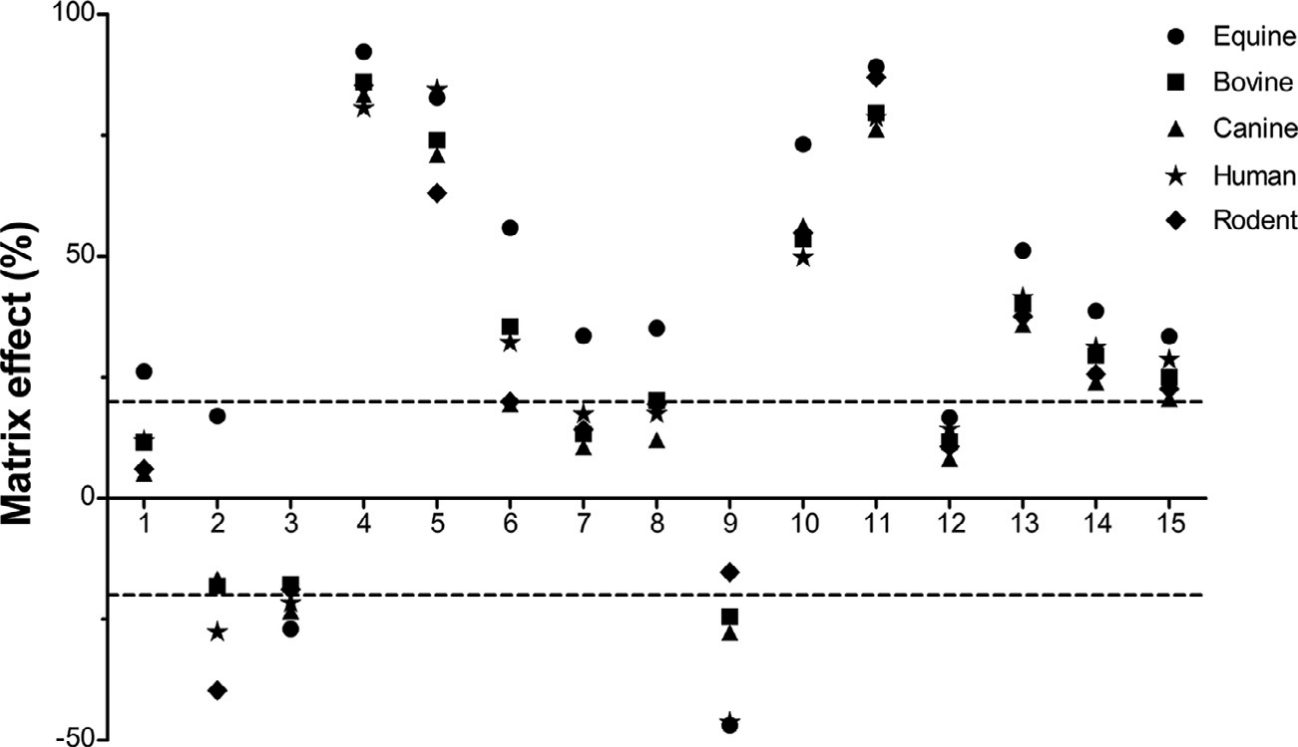
Ion suppression/enhancement results for urine matrices based on the analysis of 25 samples (*n* = 5 per species) from different sources. Values calculated as described in *Method validation* section. Negative values indicate matrix enhancement. Numbers represent analytes presented in Table 1. ––––– ±20% limit.

Method specificity has been reported previously highlighting the absence of cross talk between analytes and/or internal standards [11], whereas selectivity in this modified study was established through analysis of 161 urine samples (collected and previously tested as reported by Ventura et al. [5]) in the absence of matrix interferences. Injection of blank solvent (MeOH) following the screen positive control during every analysis was performed to monitor for carry-over, with no analyte signal in blank solvent observed. Matrix effects assessed through analysis of blank urine samples (*n* = 5 per species) of different origins spiked post-extraction at 2 × C_val_, and calculated for each analyte as the percentage difference between signals obtained when matrix extracts or a standard solution of equivalent concentration were injected, divided by the signal of the latter [16], (Fig. 2 and Supplementary data - Table S1) highlighted signal suppression for the majority of analytes, with BMS-564929 and RAD140 reporting the greatest suppression (exceeding 75% for all target species). Incorporation of affordable isotope-labelled internal standards as they become available into this method is therefore recommended with the aim of compensating for matrix effects (signal suppression/enhancement) and further improvement of accuracy and precision.

#### Detection capability (CCβ)

CCβ [14] was determined by assessing threshold value (T) and cut-off factor (Fm) [15] through analysis of equine urine (*n* = 26) from different sources, both blank and fortified at C_val_. CCβ of the screening method is validated when Fm > T [15] and then it can be concluded that CCβ is truly below the validation level. As recommended urine levels of various SARM compounds have not yet been established [17,18], C_val_ in the presented study was set as previously reported [5] at levels based on anabolic activity and comparable to that of other exogenous anabolic androgenic steroids and agents [17,19]. The developed assay enables detection of 14 SARM compounds (exception been LGD-2226 where T > Fm) in urine of all species with a false-negative rate ≤5% as stipulated in current EU legislation [14,15]. A sensitivity ≥95% at C_val_, expressed as percentage based on the ratio of samples detected as positive in true positive samples (i.e. following fortification) [20], indicates that the number of false-negative samples is truly ≤5%. Adequate low detection limits, estimated at a signal-to-noise ratio (*S/N*) of at least three measured peak-to-peak, were accomplished for all SARMs of interest excluding BMS-564929 in equine urine (eLOD 1.5 ng mL^−1^, Table 3). Absolute recoveries measured and recorded for all compounds within each analytical run aimed to verify assay performance during routine analysis (54-97%, Supplementary data – Table S1 and Fig. S2). As reported previously [5], relative cut-off factor (RFm), expressed as percentage based on the ratio of the Fm and the mean response of fortified samples, was determined for each analyte (Table 3), and during routine analysis should be applied to screen positive controls (QC samples).

**Table 3:** Validation results for fortified equine urine samples (*n* = 26).

No	Analyte	Transition (*m/z*)	eLOD^b^ (ng mL^−1^)	C_val_^c^ (ng mL^−1^)	CCβ	Relative cut-off factor (RFm)^d^ (%)	Sensitivity^e^ (%)
1	AC-262536	279.2 > 195.0	0.03	1	<C_val_	69	100
2	Andarine (S-4)^a^	440.2 > 150.0	0.06	2	<C_val_	76	96
3	Bicalutamide^a^	429.2 > 255.0	0.02	1	<C_val_	74	96
4	BMS-564929	306.1 > 96.0	1.5	5	<C_val_	66	100
5	GLPG0492	390.2 > 118.0	0.09	1	<C_val_	60	96
6	LGD-2226	393.1 > 241.1	0.18	1	>C_val_	N/A	N/A
7	LGD-4033	337.1 > 267.2	0.007	1	<C_val_	64	96
8	Ly2452473	375.2 > 272.1	0.002	1	<C_val_	17.2	96
9	Ostarine (S-22)^a^	388.1 > 118.0	0.004	1	<C_val_	75	96
10	PF-06260414	303.1 > 232.1	0.04	1	<C_val_	69	100
11	RAD140	394.1 > 223.1	0.05	1	<C_val_	37.1	96
12	S-1^a^	401.1 > 261.1	0.01	1	<C_val_	93	96
13	S-6^a^	435.1 > 145.0	0.21	1	<C_val_	34.0	100
14	S-9^a^	417.2 > 261.2	0.08	1	<C_val_	46.6	100
15	S-23^a^	415.2 > 145.0	0.11	1	<C_val_	53	100

a Values calculated response-based.

b Estimated LOD (*S/N*≥3).

c Screening target concentration.

d Calculated as percentage based on the ratio of the cut-off factor and the mean response of fortified samples.

e Expressed as percentage based on the ratio of samples detected as positive in true positive samples, following fortification.

#### Extension of validation to bovine, canine, human and rodent urine

The ruggedness study included animal species as a factor potentially impacting results, thus an extension of the initial validation in equine urine was performed with bovine, canine, human and rodent urine (by testing urine from different sources, *n* = 5 per species, both blank and fortified at C_val_ as per equine urine), providing sensitivity as highlighted in Table 4. Accordingly, the method is seen to be applicable to these additional species, with the same CCβ values for all analytes as per equine urine. Furthermore, the ruggedness study, executed on a different day and by a different operator [15], reported correct classification of all analysed urine, with 15 blank samples (*n* = 5 per species) all “screen negative” and corresponding fortified (C_val_) samples all “screen positive” (i.e. exceeding the cut-off factor).

**Table 4:** Validation results for fortified bovine, canine, human and rodent urine samples (*n* = 5 per species).

No	Analyte	eLOD^b^ (ng mL^−1^)	C_val_^c^ (ng mL^−1^)	CCβ	Sensitivity^d^ (%)
1	AC-262536	0.02	1	<C_val_	100
2	Andarine (S-4)^a^	0.05	2	<C_val_	100
3	Bicalutamide^a^	0.006	1	<C_val_	100
4	BMS-564929	0.19	5	<C_val_	100
5	GLPG0492	0.12	1	≤C_val_	95
6	LGD-2226	0.04	1	>C_val_	N/A
7	LGD-4033	0.004	1	<C_val_	100
8	Ly2452473	0.002	1	<C_val_	100
9	Ostarine (S-22)^a^	0.005	1	≤C_val_	95
10	PF-06260414	0.05	1	<C_val_	100
11	RAD140	0.09	1	<C_val_	100
12	S-1^a^	0.006	1	<C_val_	100
13	S-6^a^	0.04	1	<C_val_	100
14	S-9^a^	0.02	1	<C_val_	100
15	S-23^a^	0.02	1	<C_val_	100

a Values calculated response-based.

b Estimated LOD (*S/N*≥3).

c Screening target concentration.

d Expressed as percentage based on the ratio of samples detected as positive in true positive samples, following fortification.

#### Application to real samples

**Fig. 3:**
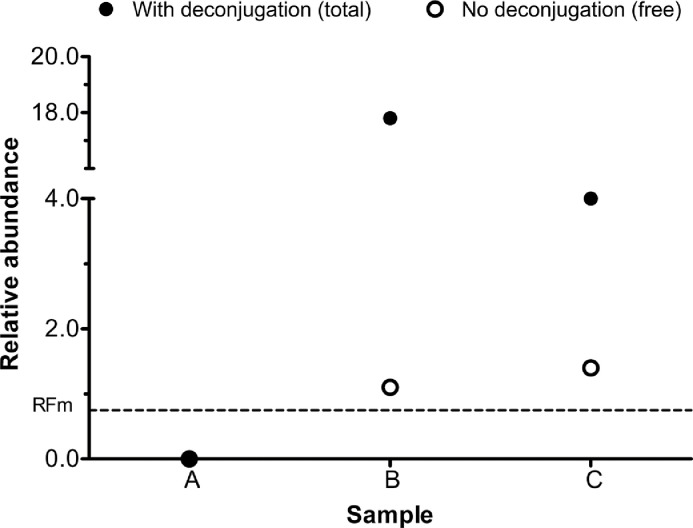
Relative abundance of ostarine (S-22) excreted in bovine urine samples collected before (A), and 2 h (B) and 3 days (C) after treatment employing assay with/without [5] deconjugation step. Relative abundance based on positive QC samples fortified at 1 ng mL^−1^ (mean, *n* = 3). ––––– Relative cut-off factor (RFm).

Bovine urine collected from a two months old steer calf orally administered 200 mg of ostarine (S-22) as described previously [6] were assayed employing the developed method. Three samples were tested blindly in triplicate and each was assigned correctly, with one sample screened negative (A - collected prior to SARM treatment), and the remaining two samples screened positive (collected B - 2 h and C - 3 days, respectively, post-ostarine (S-22) administration) – Fig. 3 and Supplementary data – Fig. S3. The current findings are in agreement with ostarine urinary concentration results (following UHPLC-MS/MS in-house validated analysis, CCα 0.25 µg L^−1^) reported by de Rijke et al. [6]. Fig. 3 depicts free [5] and total ostarine residues detected in tested bovine urine samples, whereas total form represents the sum of free ostarine and ostarine liberated within enzymatic hydrolysis step from respective conjugates. A deconjugation step included in the method led to significantly increased relative abundance of ostarine, namely 16.2-fold in sample B and 2.9-fold in sample C, respectively. Additionally, equine, bovine, canine and human urine samples (*n* = 161) have been screened employing the developed assay with hydrolysis, with no tested samples reporting detectable levels of SARM compounds.

### Concluding remarks

The current study describes the simultaneous monitoring of 14 SARMs in hydrolysed urine from equine, canine, human, bovine and rodent *via* an UHPLC-MS/MS-based semi-quantitative screening developed to incorporate an enzymatic hydrolysis step into a previously established protocol [5]. The method was validated in accordance with criteria stipulated in relevant legislation and demonstrates required sensitivity at ≥95% [14,15] with CCβ values determined at 1 ng mL^−1^, except for andarine (2 ng mL^−1^) and BMS-564929 (5 ng mL^−1^). The analysis of incurred samples highlighted the diagnostic capability of the presented method to detect a total form of emerging SARMs in urine matrix. This modified assay can serve as an effective approach to reveal illicit SARM use in urine from animal and human sport animals as well as food-based livestock.

## Declaration of Competing Interest

There are no conflicts of interest to declare.

## Appendix Supplementary materials

Image, application 1
