# Supplementary material for: Enhanced UHPLC-MS/MS screening of selective androgen receptor modulators following urine hydrolysis
Source: MethodsX. 2020 May 21;7:100926. doi: 10.1016/j.mex.2020.100926 (PMC7286957; doi:10.1016/j.mex.2020.100926)
Supplement: Supplementary file 1 [file mmc1.docx]

Supplementary data:

**Enhanced UHPLC-MS/MS screening of selective androgen receptor modulators following urine hydrolysis**

Anna Gadaj^a,b*^, Emiliano Ventura^a*^, Jim Healy^c,d^, Francesco Botrè^e^, Saskia S. Sterk^f^, Tom Buckley^g^ and Mark H. Mooney^a^

^a^ Institute for Global Food Security, School of Biological Sciences, Queen’s University Belfast, BT9 5DL, United Kingdom

^b^ Chemical and Immunodiagnostic Sciences Branch, Veterinary Sciences Division, Agri-Food & Biosciences Institute (AFBI), Stoney Road, Belfast BT4 3SD, United Kingdom

^c^ Laboratory, Irish Greyhound Board, Limerick Greyhound Stadium, Ireland

^d^ Applied Science Department, Limerick Institute of Technology, Moylish, Limerick, Ireland

^e^ Laboratorio Antidoping, Federazione Medico Sportiva Italiana, Italy

^f^ Wageningen Food Safety Research, Wageningen University & Research, European Union Reference Laboratory, Wageningen, the Netherlands

^g^ Irish Diagnostic Laboratory Services Ltd., Johnstown, Co. Kildare, W91 RH93, Ireland

*Corresponding author: anna.gadaj@afbini.gov.uk (A. Gadaj); eventura01@qub.ac.uk, emiliano.ventura@outlook.it (E. Ventura)

**Table S1**

Recovery and matrix effect data.

| **No** | **Analyte** | **Recovery (%)^a^** | **RSD (%)^a^** | **Ion suppression/enhancement (%) ± SD (%) in matrix^b^** | | | | |
| --- | --- | --- | --- | --- | --- | --- | --- | --- |
|  |  |  |  | **Equine** | **Bovine** | **Canine** | **Human** | **Rodent** |
| 1 | AC-262536 | 86 | 12.1 | 26.2 ± 5.8 | 11.6 ± 7.2 | 5.2 ± 4.5 | 11.9 ± 8.7 | 6.1 ± 3.6 |
| 2 | Andarine (S-4) | 97 | 8.5 | 17.0 ± 10.1 | -18.1 ± 13.7 | -16.6 ± 13.1 | -27.6 ± 10.6 | -39.7 ± 5.8 |
| 3 | Bicalutamide | 92 | 4.9 | -27.0 ± 4.1 | -17.8 ± 10.2 | -23.3 ± 11.8 | -21.6 ± 20.5 | -18.0 ± 9.5 |
| 4 | BMS-564929 | 91 | 8.6 | 92 ± 2.9 | 86 ± 5.1 | 83 ± 3.7 | 81 ± 10.7 | 85 ± 3.3 |
| 5 | GLPG0492 | 93 | 8.5 | 83 ± 3.9 | 74 ± 16.4 | 71 ± 7.5 | 85 ± 7.5 | 63 ± 7.9 |
| 6 | LGD-2226 | 72 | 30.8 | 56 ± 10.6 | 35.5 ± 8.9 | 19.5 ± 7.2 | 32.2 ± 22.9 | 20.0 ± 4.9 |
| 7 | LGD-4033 | 83 | 7.3 | 33.6 ± 6.0 | 13.3 ± 5.9 | 10.6 ± 6.3 | 17.4 ± 17.6 | 14.2 ± 4.3 |
| 8 | Ly2452473 | 85 | 12.4 | 35.2 ± 6.3 | 20.2 ± 10.8 | 12.1 ± 5.7 | 17.5 ± 11.5 | 19.4 ± 4.2 |
| 9 | Ostarine (S-22) | 88 | 10.4 | -46.9 ± 11.2 | -24.4 ± 16.3 | -27.7 ± 10.3 | -46.3 ± 7.2 | -15.3 ± 11.6 |
| 10 | PF-06260414 | 81 | 5.2 | 73 ± 3.6 | 54 ± 11.7 | 56 ± 12.0 | 49.8 ± 25.1 | 55 ± 4.9 |
| 11 | RAD140 | 96 | 13.9 | 89 ± 4.9 | 80 ± 9.3 | 76 ± 6.7 | 79 ± 8.6 | 87 ± 3.2 |
| 12 | S-1 | 85 | 4.7 | 16.7 ± 3.8 | 11.7 ± 2.1 | 8.2 ± 1.8 | 14.3 ± 14.0 | 10.7 ± 2.0 |
| 13 | S-6 | 54 | 36.9 | 51 ± 9.4 | 40.2 ± 3.4 | 35.9 ± 1.7 | 41.5 ± 16.8 | 37.6 ± 3.6 |
| 14 | S-9 | 64 | 24.9 | 38.7 ± 8.0 | 29.5 ± 2.1 | 24.0 ± 1.4 | 31.2 ± 16.5 | 25.7 ± 3.1 |
| 15 | S-23 | 67 | 22.8 | 33.5 ± 7.8 | 25.0 ± 2.4 | 20.6 ± 1.6 | 28.7 ± 17.4 | 22.6 ± 3.1 |

^a^ Recovery of SARMs in urine was determined by comparing results from fortified samples to those of negative samples spiked post-extraction at the screening target concentration (C_val_). Recovery is based on data collected from 13 analytical runs.

^b^ Ion suppression/enhancement results for urine matrices are based on the analysis of 25 samples (*n* = 5 per species) from different sources. Values calculated as described in *Method validation* section. Negative values indicate matrix enhancement.

**Fig. S1.** Average absolute recoveries of SARMs (and standard deviations, shown by error bars) obtained applying 50 mmol L^-1^ aq. NH_4_OH pH 10.5 and 0.1 mol L^-1^ carbonate buffer pH 9.5 in equine urine samples (*n* = 3) fortified at 1 ng mL^-1^ excluding andarine (2 ng mL^-1^) and BMS-564929 (5 ng mL^-1^).

**Fig. S2.** Average absolute recoveries of SARMs in urine (and standard deviations, shown by error bars) determined by comparing results from fortified samples to those of negative samples spiked post-extraction at the screening target concentration (C_val_). Recovery is based on data collected from 13 analytical runs (*n* = 5 equine, *n* = 2 bovine, *n* = 1 canine, *n* = 2 human and *n* = 3 rodent urine).

**(a) (b) (c)**


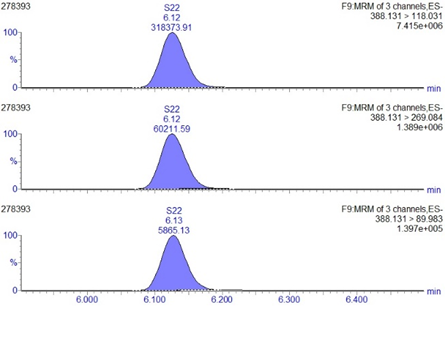
**
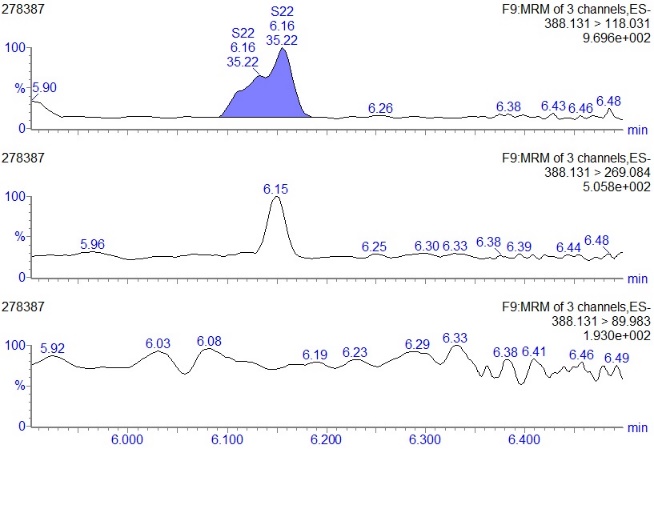

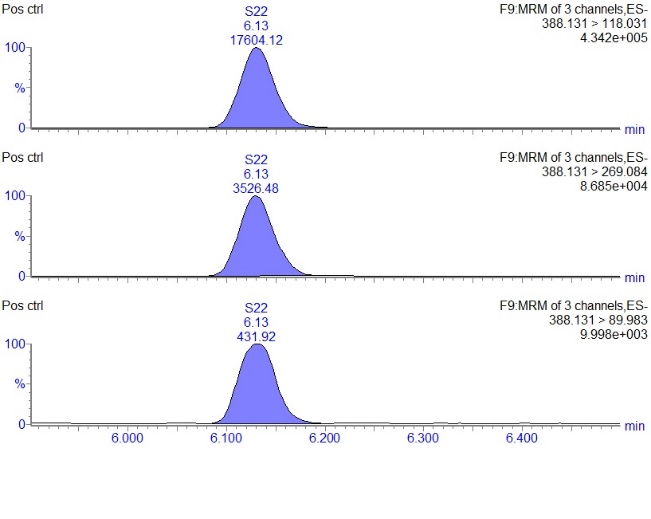
**

**Fig. S3.** UHPLC-MS/MS traces of (a) blank bovine urine sample fortified at 1 ng mL^-1^ with ostarine (S-22), (b) bovine urine sample screened negative (collected prior the treatment), and (c) bovine urine sample screened positive (collected 2 h post-administration of ostarine).
